# Diffusion of Myosin V on Microtubules: A Fine-Tuned Interaction for Which E-Hooks Are Dispensable

**DOI:** 10.1371/journal.pone.0025473

**Published:** 2011-09-26

**Authors:** Dennis Zimmermann, Basma Abdel Motaal, Lena Voith von Voithenberg, Manfred Schliwa, Zeynep Ökten

**Affiliations:** Institute for Anatomy and Cell Biology, Ludwig-Maximilians-University of Munich, Munich, Germany; University of Massachusetts Amherst, United States of America

## Abstract

Organelle transport in eukaryotes employs both microtubule and actin tracks to deliver cargo effectively to their destinations, but the question of how the two systems cooperate is still largely unanswered. Recently, in vitro studies revealed that the actin-based processive motor myosin V also binds to, and diffuses along microtubules. This biophysical trick enables cells to exploit both tracks for the same transport process without switching motors. The detailed mechanisms underlying this behavior remain to be solved. By means of single molecule Total Internal Reflection Microscopy (TIRFM), we show here that electrostatic tethering between the positively charged loop 2 and the negatively charged C-terminal E-hooks of microtubules is dispensable. Furthermore, our data indicate that in addition to charge-charge interactions, other interaction forces such as non-ionic attraction might account for myosin V diffusion. These findings provide evidence for a novel way of myosin tethering to microtubules that does not interfere with other E-hook-dependent processes.

## Introduction

Efficient long-range intracellular transport of organelles is powered by processive motors of the kinesin, dynein and myosin superfamilies [1], [2]. Studies over the past two decades have also shown a coordinated interplay between both microtubule- and actin-based transport systems [3], [4], [5], [6]. For example, in extracts of the squid giant axon, a vesicle moving along a microtubule can suddenly leave its track and continue to move on an invisible track believed to be an actin filament [7]. In fish and amphibian melanophores, pigment granules (melanosomes) are transported on microtubules towards the cell periphery by kinesin-2 where their movement on actin filaments is driven by myosin V (myo V) [8], [9], [10], [11]. To date, the regulation of this crosstalk remains elusive.

Studies by Ali *et al.* [12] have added a further facet to the interaction between actin and microtubule motor systems, where myo V was found to switch from an actin filament onto an intersecting microtubule followed by one-dimensional diffusive motion over large distances *in vitro*. In previous studies it was shown, that a positively charged structural element (loop 2) of the myo V catalytic head stabilizes the weakly bound state of myo V to negative patches on the actin filament via electrostatic interactions [13], [14], [15], [16]. Likewise, several kinesins interact via a positively charged motor domain structure (K-loop) [17], [18], [19], [20], [21], [22] with the negatively charged C-Terminus (E-hook) of microtubules extending from the surface [23], [24]. Based on these findings, and to explain the observed myo V-microtubule interaction, the authors proposed that the diffusion of myo V on microtubules is mediated by an electrostatic interaction of the positively charged loop 2 and the negatively charged E-hooks. To understand the molecular basis of this interaction three major questions have been addressed here: Does indeed the charge of loop 2 contribute to microtubule binding? Or does rather the amino acid composition of loop 2 make the difference? And most importantly, after the initial binding to the microtubule, what biophysical feature enables myo V to start diffusing along the filament?

We used single molecule Total Internal Reflection Fluorescence Microscopy (TIRFM) to characterize the association and diffusion of myo V mutants containing negatively charged loop 2 motifs on microtubules. The previously proposed electrostatic model predicts that myo V mutants containing a net negative charge on their loops would cease to interact with the negatively charged microtubules. Surprisingly, myo V diffusion on microtubules is neither determined nor limited by the charge of loop 2 as both, the positively charged *Wildtype* myo V and the negatively charged loop 2 mutants, bind to and diffuse on microtubules. Most strikingly, neither for the initial association nor for the subsequent diffusion of myo V along microtubules E-hooks are required. Additional analysis of the microtubule binding and diffusion behavior of our oppositely charged constructs suggests that in addition to charge-charge interactions between myo V and the microtubule also non-ionic (e.g. van-der-Waals) attraction co-determines the interaction between myo V and microtubules, while hydrophilic effects by loop 2 merely play a subordinate role in facilitating diffusion on intact microtubules.

## Results

### Design of the myosin V loop 2 mutants

Loop 2 of myo V is a well-defined region of ∼44 amino acids near the so-called 50/20 kDa junction of the head domain. It has been implicated in binding the motor domain to actin [14], [15] and recently has been suggested to also mediate the interaction of myo V with microtubules [12]. It is surface-exposed and exhibits a net positive charge of +5.

**Figure 1:**
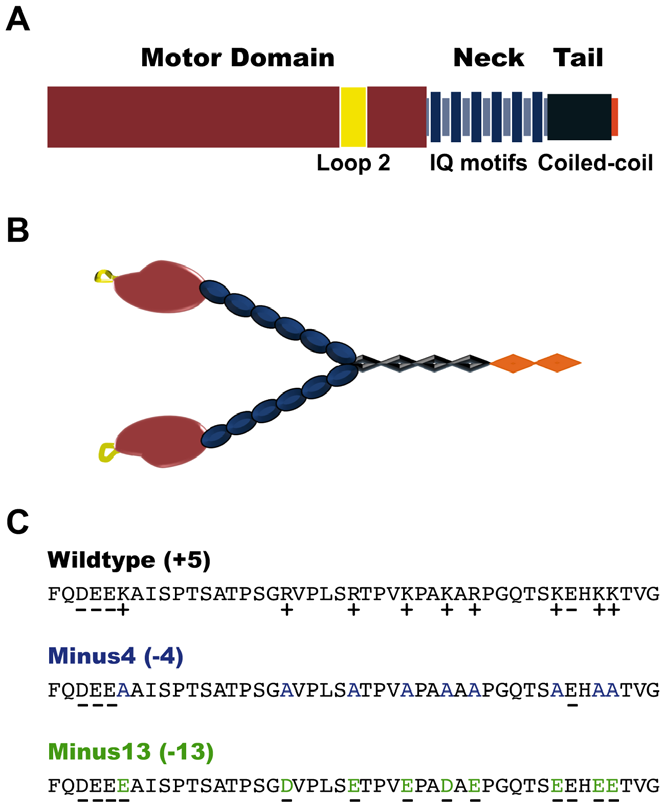
Schematic representations of the myosin V loop 2 constructs. (A) Schematic diagram depicting the domain structure of myo V with its surface-exposed loop 2 shown in yellow. The C-terminal GCN4 motif ensures dimerization. (B) Schematic domain model of truncated myo V in its homodimeric form. The regions are color-coded for each structural motif. The same set of colors is used in A and B. (C) Comparison of the loop 2 sequences from myo V *Wildtype*, *Minus4* and *Minus13*. Mutants *Minus4* (blue) and *Minus13* (green) were designed by altering positively charged amino acids (lysines and arginines) to alanine and glutamic/aspartic acid, respectively. (+) and (−) indicate positively and negatively charged amino acids, respectively. The net charge of each construct is indicated in brackets next to the respective construct.

To dissect the contribution of the myo V loop 2 on the interaction with microtubules, we have generated two heavy mero myosin (HMM)-like myo V mutants with surplus net negative charge by substituting the positively charged residues of loop 2 with either alanine (*Minus4*, four negative net charges) or glutamic and aspartic acid (*Minus13*, thirteen negative net charges) (Figure 1).

### A negative net charge on loop 2 impairs the myosin V interaction with actin filaments

To assess the functionality of our constructs, we performed ATPase activity and *in vitro* single molecule motility measurements on actin. The *in vitro* single-molecule velocity and runlength measurements (Figure S1, A-B and Table S1), as well as the ATPase activity values of HMM-like *Wildtype* myo V (Figure S1C and Table S1) were in good agreement with those reported previously [13], [15], [25], [26], [27]. Several previous studies showed that decreasing the positive net charge on loop 2 leads to a decreased affinity for actin [14], [15], [28]. In line with that, the ATPase activity on F-actin was significantly decreased (*Minus4*) or abolished (*Minus13*) for the myo V constructs carrying a negative net charge on their loop 2. (Figure S1C and Table S1). Accordingly, *in vitro* motility studies did not yield any single molecule runs for those two constructs. Thus only a positively charged loop 2 promotes a productive interaction with actin.

### Loop 2 is not the prime determinant of the interaction between myosin V and microtubules

**Figure 2:**
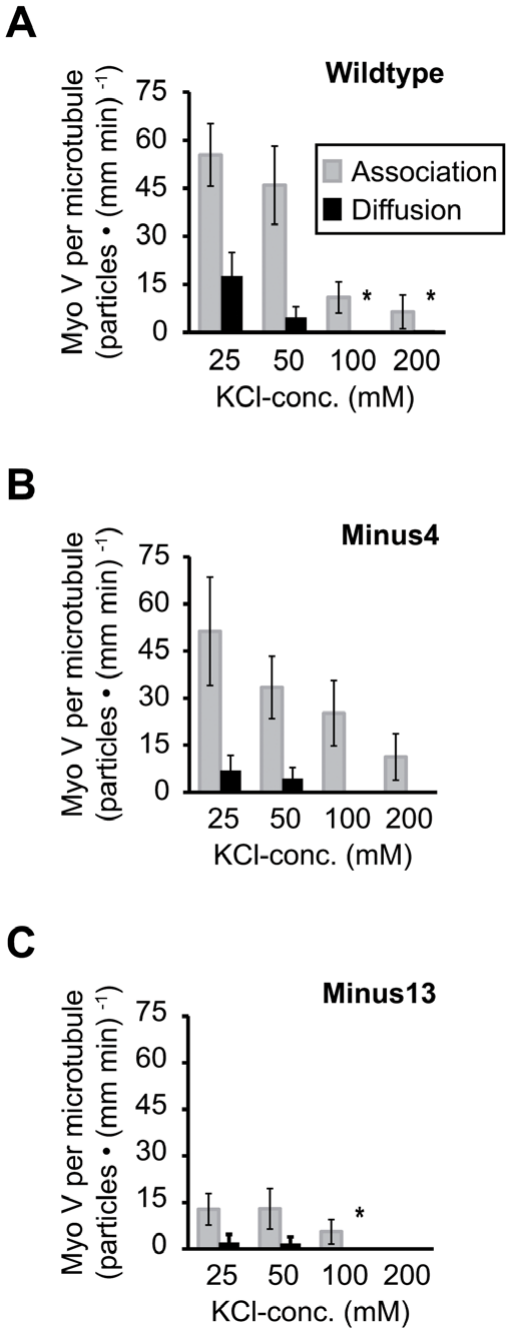
Interaction of myosin V with microtubules under increasing ionic strength conditions. TIRFM movie sequences of single-molecule experiments with 100 nM Cy3-labeled myo V *Wildtype* (A), *Minus4* (B) and *Minus13* (C) on Atto488-labeled microtubules under increasing salt concentrations (i.e. 25, 50, 100 and 200 mM KCl), were analyzed for microtubule-association and subsequent diffusion over a 70-seconds time period. For this, the total number of microtubule-associated myo V particles (shaded) and the number of diffusing particles (solid) per unit length of the microtubules and time of measurement are plotted as a function of KCl concentration. (*) indicates that in those cases only one event in total was counted. Error bars represent mean ± confidence interval (α = 0.95).

To uncover the role of electrostatic interactions via myo V loop 2, we assessed the effects of increasing KCl-concentrations on the *Wildtype* and loop 2 mutants by quantifying the number of microtubule-interacting myo V molecules. Here, we discriminated between microtubule-colocalization events (defined as *association* events) and events where association was followed by subsequent diffusion (defined as *diffusion* events). To this end, the number of events per microtubule unit length and time period was determined at KCl concentrations of 25, 50, 100 and 200 mM. An increase in ionic strength significantly decreased microtubule association of *all* three constructs (gray bars in Figure 2, A–C and Table S2). The *Minus13* mutant, in particular, showed the most dramatic reduction of microtubule association, even at moderate ionic strength (Figure 2C). We attribute this behavior to a pronounced repulsion between the highly negatively charged motor loop and the negatively charged surface of the microtubule [29]. Although this finding indicates that the surface-exposed loop 2 may indeed participate in myo V's affinity for microtubules, for two obvious reasons, the observed attraction cannot be primarily attributed to simple charge-charge interactions between the negatively charged microtubule and the normally positively charged loop 2: first, the positively *and* the negatively charged loop 2 constructs display similar salt-dependent decrease in microtubule association (gray bars in Figure 2, A–C); second, despite carrying opposite net charges on their loop 2, *Wildtype* and *Minus4* show comparable levels of association at 25 mM salt (Table S2).

Taken together, our results now make testable predictions for myo V's capability to diffuse on the microtubule. If, as previously proposed [12], the interaction between myo V and microtubules were exclusively dependent on electrostatic forces, only *Wildtype* myo V (positive loop 2 net charge) would be expected to show diffusion on microtubules. However, if this interaction depends either on additional attraction forces or/and domain regions other than the proposed loop 2, the two negatively charged constructs, *Minus4* and *Minus13*, should as well diffuse along the negatively charged microtubule lattice.

Indeed, irrespective of their net charge, all three myo V constructs showed diffusion along microtubules (Figure 2, black bars). Due to the clear salt-dependent interactions shown here, charge-charge interactions are likely to dominate the binding of myo V to microtubules. Notably, not only at low-salt conditions but also, at 100 mM KCl (*Wildtype* and *Minus13*) and 200 mM KCl (*Wildtype*) did we observe diffusion (Figure 2, black bars, Table S2 and Video S1). Hence it can be concluded that electrostatic attraction mediated by loop 2 cannot be considered the sole molecular determinant for microtubule association. Therefore, it cannot be ruled out that other attraction forces (e.g. non-ionic) co-determine myo V's affinity toward microtubules.

### All myosin V loop 2 charge mutants show unperturbed diffusion on microtubules

**Figure 3:**
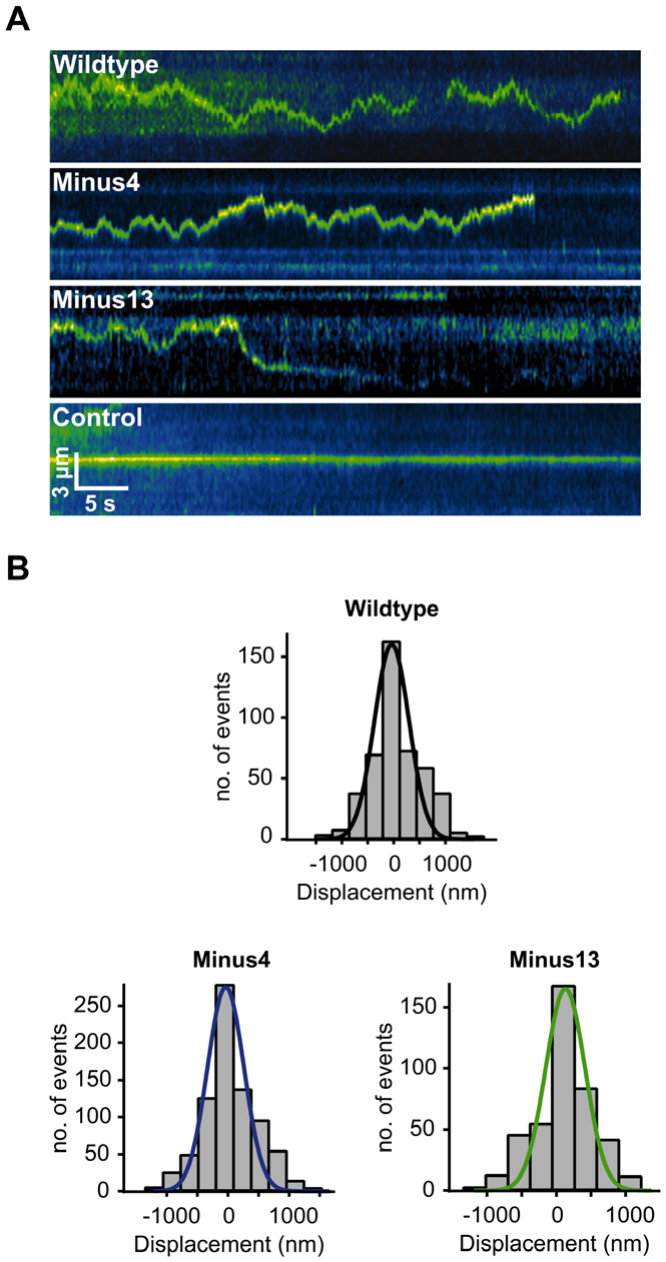
One-dimensional diffusion of myosin V loop 2 constructs on microtubules. (A) Kymographs of sequential frames depicting diffusive movement of single Cy3-labeled myo V *Wildtype*, *Minus4* and *Minus13* molecules (top to bottom, pseudo-colored green) on Atto488-labeled microtubules in 25 mM KCl. Microtubules are not shown for this purpose. Control represents a stationary, non-diffusing motor molecule on the microtubule. (B) TIRFM movie sequences of single myo V molecules on microtubules in 25 mM KCl were analyzed and then plotted as a displacement histogram. A single Gaussian (solid color-coded lines) was fitted to the data using equation 1 (Methods). From the obtained fit, the variance *σ* was used to calculate the diffusion coefficient *D* according to the equation, *D* = *σ*/2*t*, where *t* is the time interval between images, resulting in *D_Wt_* = 0.113 µm^2^/s (n = 464), *D_Minus4_* = 0.089 µm^2^/s (n = 801) and *D_Minus13_* = 0.081 µm^2^/s (n = 425). Black, blue and green color-coded fit-lines depict the Gaussian fit for the individual displacement distribution of myo V *Wildtype*, *Minus4* and *Minus13*, respectively.

Next, we characterized the behavior of the diffusion events for myo V *Wildtype* and the loop 2 mutants on microtubules (Figure 3A, Videos S2 and S3). Diffusion of *Wildtype* yielded a diffusion constant (*D* = 0.113 µm^2^/s) which is in good agreement with previous work [30] (Table 1 and Video S2). Strikingly, our analysis demonstrates that the *D*-values for the loop 2 mutants were similar compared with *D* of *Wildtype* (Table 1). Additional support came from the linear increase in mean square displacement (*MSD*) over time (Figure S2, B–D) [31], [32], [33]. As expected, by calculating *D* from the obtained slopes in the *MSD*-plots, we could confirm the validity of the Gaussian-based *D*-value calculations.

**Table 1:** Key parameters of diffusion of myosin V (*Wildtype*) and two myosin V loop 2 mutants.

	Diffusion coefficient (*D*) (µm^2^ s^−1^)	Maximum speed (*V_max_*) (µm s^−1^)	Scan distance (*x_scan_*) (µm)	Association time (*t_A_*) (s)	n Diffusion events	n Displacement events
MyoV (Wildtype)	0.113±0.004***	1.04±0.06**	2.21±0.27*	14.1±0.5***	31	464
MyoV (Minus4)	0.089±0.003***	0.99±0.06*	2.95±0.28*	23.8±3.2 ***	35	801
MyoV (Minus13)	0.081±0.003***	0.84±0.06**	2.07±0.23*	16.2±0.8**	32	425
*on S-microtubules*						
MyoV (Wildtype)	0.226±0.006***	1.21±0.09**	3.02±0.34*	12.4±2.7*	20	327

Diffusion coefficients (*D*) were measured from the variance of the Gaussian fit function of the displacement histogram (Figure 3B). Values for maximum speed (*V_max_*) reflect the average maximum displacement along a microtubule during one frame interval (i.e., 5 frames per s) and were calculated from the respective number of analyzed diffusion events. Scan distances (*x_scan_*) were calculated as the distance between the two extreme positions the motor has scanned on the microtubule during one diffusion event. Association times (*t_A_*) represent the total time a myo V molecule spent on the microtubule during one diffusion event. Mean values for *t_A_* were measured from the exponential fit of the plotted histogram of single *t_A_*-values (Figure S3). Diffusion events are defined as events during which the myo V molecule has bound to the filament and subsequently started diffusing. Diffusion was quantified by single displacement measurements. Parameters of myo V diffusion on subtilisin-treated microtubules (*S-microtubules*) for *Wildtype* are shown in the bottom part. All parameters were obtained from experiments in 25 mM KCl. *n* represents the total number of analyzed diffusion and displacement events. Values represent mean ± S.E.M. Statistical significance at *P>0.05, **P<0.05 and ***P<0.005 vs. *Wildtype* was determined using Student's t-Test.

The diffusion-derived single displacements of all three constructs distributed as zero-centered Gaussians (Figure 3B), as expected for one-dimensional Brownian motion [34]. Zero net displacement was also confirmed by boxplots (Figure S2A). During diffusion, the time of association with the microtubule (*t_A_*) for all four constructs distributed exponentially (Figure S3, A–C). Individual mean values of *t_A_* (Table 1) ranged from 14.1 s to 23.8 s, with *t_A_* of *Wildtype* being the shortest. Finally, the average distance scanned by the respective constructs during diffusional events did not vary significantly amongst *Wildtype* and *Minus13* and was only slightly larger for *Minus4* (Table 1).

As expected, diffusion did not require ATP [12], [31], [35]. Control experiments confirmed that the observed diffusion was not caused by the antibody that coupled the motor to the fluorophore, nor did the antibody display any specific association with microtubules.

In line with the above myo V-microtubule *association* analysis, the *diffusion* analysis performed here strongly supports the notion that the interaction between the motor and the microtubule is not restricted to probable loop 2-conveyed electrostatic forces only. Most notably, Minus13, a construct carrying 13 negative net charges on its loop 2, shows a highly similar diffusion behavior as the positively charged *Wildtype* (Figure 3B).

We next assessed the proposed role of the negatively charged E-hooks [12] in tethering myo V to the microtubule surface.

### Myosin V diffusion takes place without the help of E-hooks

**Figure 4:**
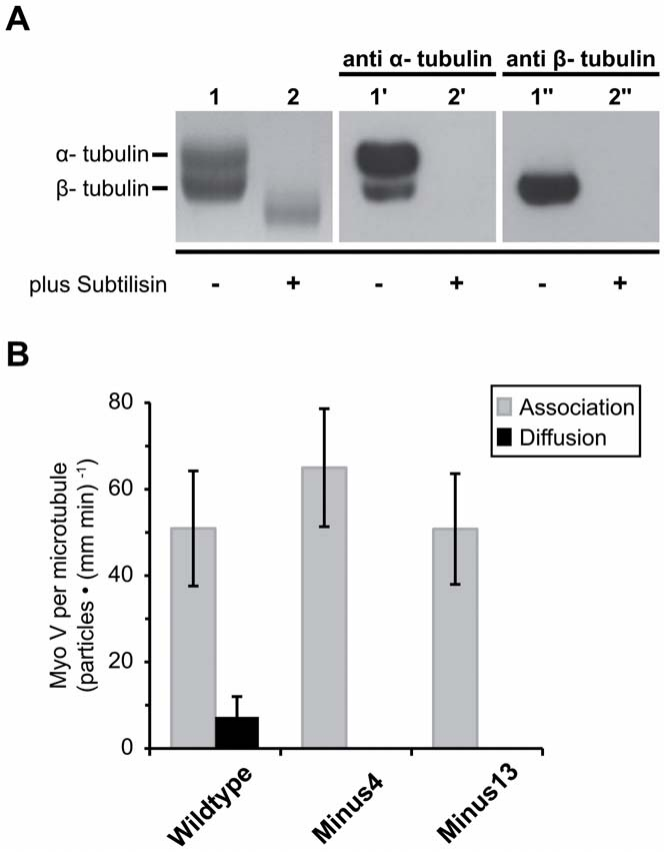
Interaction of myosin V with S-microtubules. (A) Removal of the Carboxy-terminal E-hook from microtubules. (Left panel) SDS/12% PAGE gel of untreated microtubules (lane 1), and after subtilisin-treatment (lane 2). (Middle and right panel) Western blots of these two lanes with anti-α or anti-β tubulin antibodies. Subtilisin-treatment resulted in the complete loss of epitope reactivity, hence complete E-hook removal can be assumed. (B) TIRFM movie sequences of single-molecule experiments with 100 nM Cy3-labeled myo V *Wildtype*, *Minus4* and *Minus13* on Atto488-labeled S-microtubules, in 25 mM KCl were analyzed for microtubule-association and subsequent diffusion over a 70-seconds time period. The total number of S-microtubule-associated and diffusing myo V particles per unit length of the microtubules and time of measurement is plotted as category plot for the respective myo V constructs. Error bars represent mean ± confidence interval (α = 0.95).

E-hooks give rise to a pronounced negatively charged mantle around the microtubule [34]. They have long been known to facilitate one-dimensional diffusion of a number of microtubule-associated proteins by tethering them electrostatically to microtubules [17], [18], [20], [36], [37]. Besides the fact that the observed electrostatic attraction towards microtubules is not mediated by positive charges on loop 2 of myosin, additional non-ionic attraction forces might also be involved in myo V's interaction with microtubules. To test if the negatively charged E-hooks play a substantial role in microtubule association and diffusion of the myo V constructs, we made use of limited proteolysis by subtilisin to generate S-microtubules that lack the E-hook [23], [31]. E-hook removal was confirmed by SDS-PAGE and Western blot analysis (Figure 4A) [17], [23], [38].

In favor of the non-ionic attraction model, the absence of E-hooks does not interfere with the ability of *Wildtype* to associate with microtubules (Figure 4B and Table S3). Moreover, for all three loop 2 constructs the number of associated motors per microtubule unit length leveled off (gray bars in Figure 4B and Table S3). For the most negative *Minus13* construct, E-hook removal resulted in a significant increase in microtubule association by 75% (p<0.005, Table S2 vs. Table S3).

**Figure 5:**
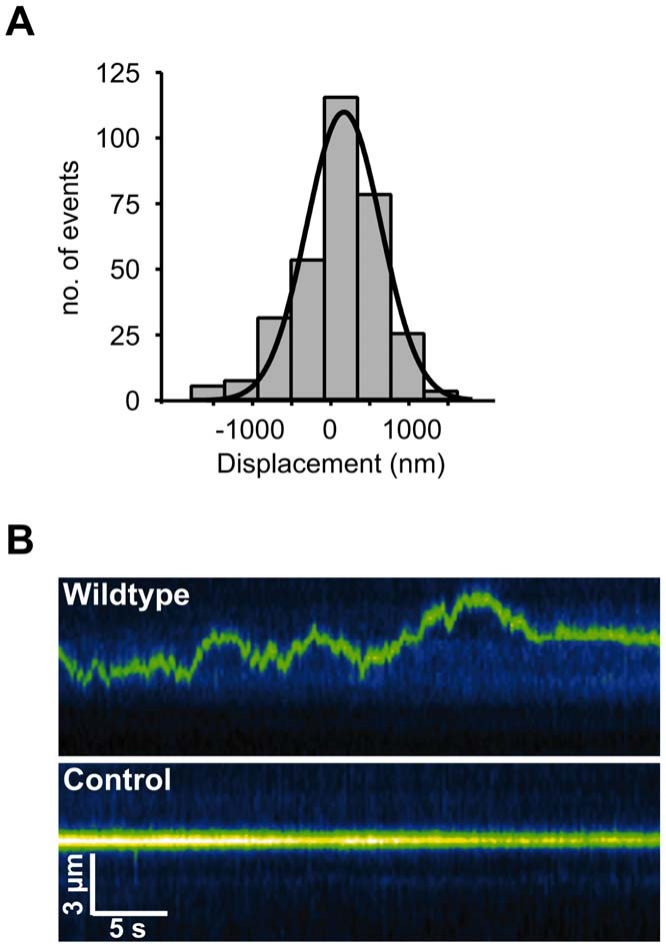
One-dimensional diffusive motion of myosin V on microtubules lacking the E-hook. (A) The displacement between successive image frames of diffusive movements for myo V *Wildtype* on S-microtubules was determined. TIRFM movie sequences of 100 nM Cy3-labeled myo V *Wildtype* on Atto488-labeled microtubules were analyzed and plotted as a displacement histogram. The diffusion coefficient *D* was calculated according to the equation, *D* = *σ*/2*t*, where *t* represents the time interval between images and *σ* the variance. *σ* was obtained from the Gaussian fit (solid line), resulting in *D* = 0.226 µm*^2^*/s (n = 327). (B) Kymograph of sequential frames depicting the diffusive motion of a single Cy3-labeled myo V *Wildtype* molecule (pseudo-colored green) on Atto488-labeled S-mmicrotubules (for this purpose not visualized). Control represents a stationary, non-diffusing motor molecule on the microtubule.

Most importantly, the removal of E-hooks did not interfere with the ability of *Wildtype* myo V to diffuse along S-microtubules. The diffusion behavior of *Wildtype* on S-microtubules meets all the criteria of a one-dimensional diffusion process (Figure 5A and Figure S4) [12], [31], [32], [33], [34]. Compared to untreated microtubules, with 1.21±0.09 µm/s myo V's diffusion on S-microtubules exhibits on average larger maximum displacements per frame interval (i.e., maximum speed) and shows a trend toward prolonged scan distances (3.02±0.34 µm), while the average association time of 12.4 s with microtubules remained essentially unchanged (Table 1 and Figure S4A). Notably, the diffusion coefficient *D* = 0.226 µm^2^/s was twice as high as that of *Wildtype* on untreated microtubules (Table 1 and Figure 5).

### Non-electrostatic attraction forces contribute to the interaction of myosin V with microtubules

The striking observation that irrespective of the net charge of loop 2 all three constructs bind to S-microtubules, while only *Wildtype* (Figures 4B and 5, Table S3 and Video S4) diffuses, raises the question whether besides electrostatics also additional attraction forces contribute to the interaction between myo V and microtubules. We propose that due to the E-hook removal, the formerly homogeneously negatively charged microtubule is now marked by negative and also positive surface charges [29], [39]. Hence, formerly prevalent electrostatic *repulsion* forces (negative loop 2 vs. negative E-hooks) are now eliminated and electrostatic *attraction* forces (negative loop 2 vs. positive patches on the microtubule surface) are now free to exert their effects. The absence of the ∼4 nm E-hook spacers would enhance additional, non-ionic attraction forces that have strong effects over short distances, thereby preventing the individual molecule from advancing from an associative to a diffusive state (as is the case for *Minus4* and *Minus13*) (Figure 4B). Under such circumstances, only constructs bearing a residual ionic repulsive capacity (positive loop 2 vs. positive patches on the microtubule surface), such as the *Wildtype*, display diffusion (Figure 4B). Taken together, we suggest that additional, non-electrostatic attraction forces contribute to myo V's interaction with microtubules.

## Discussion

Compared to motor-driven directed movement, unbiased one-dimensional diffusion is faster over short distances and does not consume energy, thus representing an efficient and supportive mechanism for intracellular transport processes [33], [35], [37], [40], [41]. Recent studies by Ali *et al.* [12], [30] suggested that one-dimensional diffusion of myo V on microtubules is based on electrostatic interaction between the positively charged loop 2 of myo V and the negatively charged E-hooks of microtubules. Here we probed the proposed model of electrostatic myo V-tethering to the microtubule via complementary charged stretches. For this myo V mutants carrying either moderate or high surplus *negative* charges on their loop 2 were generated. To gain detailed mechanistic insights into the unbiased diffusion of myo V, we distinguished between association of myo V with microtubules (i.e. binding without additional quantification of subsequent diffusion events) and diffusion *per se*. From our analyses now a more complex and multilayered picture of the interaction modes between myo V and microtubules emerges.

The observation that not only positively but also *negatively* charged loop 2 constructs displayed a salt-dependent decrease in microtubule association (Figure 2) allows the following two conclusions. First, electrostatic interaction is indeed the prevailing force mediating the association of myo V with microtubules. Second, loop 2 is not the site responsible for such attraction because the oppositely charged loop 2 constructs *Wildtype* and *Minus4* (+5 vs. −4) display equivalent salt-sensitive binding behavior. Along with the observation that the *Wildtype* and *Minus4* associate at equivalent levels with microtubules further supports the notion that loop 2 neither mediates nor maintains the interaction between myo V and microtubules (Figure 2A and 2B). Thus another charged patch (or even multiple patches) on the myo V surface, mediating the observed salt-dependent interaction with the microtubule, need to be considered. Interestingly, as soon as enough negative charges were introduced into myo V loop 2 (e.g. *Minus13*), microtubule “affinity” for myo V was significantly reduced. This finding points to strong repulsion forces arising from the evenly distributed negative charges on its loop 2 motif (Figure 2C). This effect is readily revoked after the repulsive microtubule element (i.e., E-hooks) is removed (compare Figure 2C with Figure 4B). Taken together, we suggest that the dominating force that tethers myo V to the microtubule is due to charge-charge interactions. However, those interactions are by no means mediated by loop 2. In addition to electrostatic forces, non-electrostatic forces may exert significant influence at the myo V-microtubule interface.

Most strikingly, and contrary to the predictions inferred from the electrostatic model, the charge of loop 2 neither determines nor limits the diffusion behavior of myo V. The narrow range of the diffusion constants (0.113 µm^2^/s of *Wildtype* to 0.089 µm^2^/s of *Minus13*) (Figure 3B, and Table 1) argues against a loop 2-biased charge-dependence of diffusion.

The fact that on the side of the interactor (i.e., myo V) loop 2 as the potential electrostatic tether structure is neither required for the binding to nor for the diffusion along microtubules, prompted us to dissect the potential contributions of the substrate (i.e., microtubule) to myo V association and diffusion. If indeed attraction forces other than electrostatic tethering contribute to the interaction between myo V and microtubules, then microtubule E-hooks representing the proposed electrostatic tethering structures should be dispensable. Indeed, on S-microtubules lacking E-hooks, association levels for *Wildtype* and *Minus4* remained unchanged compared to untreated microtubules (Figure 4B vs. 2A and 2B), demonstrating that E-hook-mediated tethering is not involved in microtubule association of myo V. Removing the E-hooks only affected the *Minus13* construct containing an unusually high negative charge on its surface (as discussed above).

While the capacity to interact with S-microtubules did not differ significantly, diffusion was observed exclusively with the *Wildtype* construct (Figure 4B and Table S3). In other words, on microtubules lacking E-hooks, the fraction of associated motors remains high, though fewer of the attached myo V molecules advance to the diffusive state and thus remain stationary. As soon as the nearly homogeneous mantle of negatively charged E-hooks is removed, non-ionic forces (e.g. van-der-Waals interactions) that show strong effects over short distances [reviewed in 42], [43] become a substantial attraction force. We therefore suggest that non-ionic attraction forces account for the observed decrease in diffusion for all three myo V constructs, while the overall affinity remains comparable to that of *Wildtype* myo V on untreated microtubules. In addition, when E-hooks are present, they may act as negatively charged 4 nm spacers [29], [34] that via repulsion force facilitate transitions from the stationary to the diffusion phase. Hence on microtubules that lack E-hooks, constructs containing a pronounced negatively charged loop 2 region (*Minus4* and *Minus13*) are now free to productively interact with the un-shielded positive patches on the “naked” microtubule surface [29], [39] (Figure 4B). In contrast, the *Wildtype* construct with its net positive loop 2 charge, retains some residual ionic repulsive capacity (positive loop 2 vs. positive patches on the microtubule surface) and thereby on S-microtubules still manages to advance into the diffusive state (Figure 4B and 5, Video S4).

**Figure 6:**
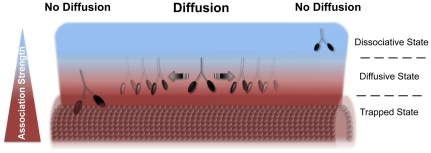
The balance between attraction forces determines the diffusive state of myosin V on microtubules. (Left part) Strong attraction forces prevent microtubule-bound myo V molecules from advancing to the diffusive state. This *Trapped State* is achieved, if in addition to electrostatic also non-ionic attraction forces become increasingly dominant (*Minus4* and *Minus13* on S-microtubules). (Middle part) Diffusion takes place if for myo V the attraction toward the microtubule is of moderate strength. This *Diffusive State* in general is achieved when attraction and repulsion outweigh each other. Two different possibilities might account for that behavior. First, electrostatic and non-electrostatic interaction forces at the myo V binding-interface are well-balanced (*Wildtype* on untreated and S-microtubules); second, strong loop 2-derived ionic attraction is dominated by ionic repulsion elements (E-hooks) on the microtubule binding-interface (*Minus4* on untreated microtubules). (Right part) Weak attraction toward microtubules prevents myo V from binding effectively to the filament, and hence diffusion becomes unlikely. This *Dissociative State* is given, if electrostatic repulsion via hydrophilic surface structures (E-hooks) becomes predominant (*Minus13* on untreated microtubules). *Red* and *blue* colors indicate strong and weak attraction forces toward the microtubule surface, respectively.

In analogy to recent findings by Minoura *et al.* [34] and based on our results, we propose the following two-phase model for diffusion of myo V on microtubules. *Phase One* is the initial association with the filament. This step represents a prerequisite for diffusion and is accomplished as long as electrostatic surface effects exerted from both, the interactor and the substrate, cause attraction rather than repulsion. *Phase Two* is the advancement to the diffusive state. This phase heavily depends on a balanced interplay between attraction and repulsion of myo V to and from the microtubule. Here, it is crucial that the strength of attraction is restricted to such an extent that the motor is free to move laterally. Simply put, strong attraction forces may bind a high number of motor molecules to the surface but they also prevent those motors from moving (*Minus4 and Minus13* on S-microtubules); conversely, weak attraction favors the diffusion along filaments, but at the same time gives the molecule a hard time to initially bind (*Minus13* on untreated microtubules). Since the dosage makes the difference, in this specific case the actual dosage of electrostatic vs. non-ionic attraction on the surface determines the strength of binding and the likelihood of moving (Figure 6).

The fact that only *Wildtype* is still capable of diffusing on S-microtubules (Figure 5) suggests that only the native form is capable of balancing the interplay between ionic and non-ionic effects, making *Wildtype* almost “immune” to harsh changes on the microtubule (Figure 6). A closer look at the diffusion behavior of *Wildtype* reveals that compared to untreated microtubules, its diffusion along S-microtubules in fact is smoother (larger single displacements), yielding an increased diffusion constant (Figure 5A and Table 1). The E-hook-independent mode of interaction displayed by myo V offers an attractive explanation for the recent observation of myo V-aided movement of kinesin. Myo V was shown to increase kinesin's run length on microtubules *in vitro* [30]. Our results point to a synergism between E-hook-independent tethering by myo V to microtubules that enhances the E-hook-dependent processive movement of kinesin.

In this respect unbiased diffusion of myo V contrasts with biased diffusion of certain kinesins such as MCAK along microtubules, which is E-hook-dependent [20], [44]. A different kinesin motor, kip3 from yeast, which belongs to the kinesin-8 class, also diffuses on microtubules, but does so without the help of E-hooks [45]. Thus different types of motors can diffuse on microtubules, but the underlying mode of interaction differs.

## Materials and Methods

### Reagents

All reagents were the highest purity commercially available and were obtained from Sigma-Aldrich (Munich, Germany), unless mentioned otherwise.

### Buffers

Buffers used in this study are defined as: Buffer A (80 mM Pipes, pH 6.9, 1 mM MgCl_2_, and 1 mM EGTA); Buffer B (25 mM imidazole, pH 7.4, 25 mM KCl, 4 mM MgCl_2_, 1 mM EGTA, and 10 mM DTT); Buffer C (Buffer B plus oxygen scavenging system composed of 1700 U/ml catalase, 26 U/ml glucose oxidase, and 0.4 mg/ml glucose); Buffer D (0.3 M KCl, 25 mM imidazole, pH 7.4, 4 mM MgCl_2_, 1 mM EGTA, and 10 mM DTT); Buffer E (Buffer B plus 5 mg/ml BSA); Buffer F (50 mM KCl, 10 mM imidazole, pH 7.0, 1 mM EGTA, 1 mM MgCl_2_, and 1 mM DTT); Buffer G (25 mM potassium acetate, 12.5 mM Aces·KOH, 2 mM magnesium acetate, 1 mM EGTA).

### Protein expression and purification

The constructs described below were cloned from the p2Bac/pFastBac-dhM5-CaM plasmid [46] and contain the first 1098 amino acids (D1098) of chicken myo Va HMM (6 IQ), with a leucine zipper fused in frame to the native coiled coil to ensure dimerization. To facilitate purification, a FLAG-tag epitope (DYKDDDDK) was introduced at the N-terminus. Myo Va was co-expressed with the human essential light chain using the Baculovirus Expression System (Invitrogen, Darmstadt, Germany) and purified as described [46]. The loop 2 mutations were introduced by custom DNA synthesis (Sloning BioTechnology, Puchheim, Germany). For myo V *Minus4*, all lysines and arginines within loop 2 were changed to alanines (K607A, R619A, R624A, K628A, K631A, R633A, K639A, K642A, K643A). For myo V *Minus13*, all lysines and arginines were substituted with glutamic or aspartic acid (K607E, R619D, R624E, K628E, K631D, R633E, K639E, K642E, K643E). Both loop 2 mutants were cloned into the p2Bac/pFastBac-dhM5-CaM plasmid. Expression and purification of the constructs were performed as described [46], [47]. The purified proteins were dialyzed for 1 h at 4°C in the absence of ATP against Buffer B (plus 50% glycerol) and stored at −20°C.

### Microtubule and F-actin preparation

Microtubules were prepared from porcine brain tubulin [48]. Labeling of tubulin with Atto488 (AttoTec, Siegen, Germany) and the polymerization of microtubules was performed as described in [49]. For the preparation of fluorescent microtubules, Atto488-tubulin was mixed with unlabeled tubulin at 1∶50 ratio.

Actin from chicken pectoralis was extracted from an acetone powder according to [50]. Purified G-actin (5 µM) was polymerized in Buffer B (plus 5 µM phalloidin) for 1 h at room temperature. For the preparation of fluorescent actin filaments, polymerization was performed in the presence of 5 µM TRITC-phalloidin.

### Fluorescent labeling of proteins

FLAG-purified myo V protein was conjugated to monoclonal anti-FLAG Cy3 antibody (#A9594 clone M2) by incubating 500 nM myo V protein with 11 µg/ml antibody at room temperature for 5 min in Buffer B and subsequent storage on ice. The mixture was further diluted in Buffer C to the desired final myo V concentration before use.

### Flow cell preparation

For single-molecule motility assays 15-µl flow cells (area 18×5 mm), covered by a nitrocellulose coverslip, were used. For assays with microtubules as the cytoskeletal track, fluorescent microtubules (1 µM) (Buffer A plus 5 µM taxol) were infused into the flow cell and incubated for 3 min, followed by a wash with Buffer A (plus 5 µM taxol, and 0.7 mg/ml casein). 100 nM fluorescent-labeled myo V in Buffer C was added to the flow cell and incubated for 1 min. For experiments in which different ionic strength-conditions were applied to the flow cell, the required volume of KCl (1 M) was added to Buffer C yielding a final assay concentration of 50, 100 or 200 mM KCl.

For assays in which actin filaments served as cytoskeletal tracks, flow cells were pre-incubated with 0.1 mg/ml *N*-ethyl maleimide (NEM)-modified heavy mero myosin (HMM) [51] in Buffer D for 3 min, rinsed with Buffer B, incubated with 0.1 µM TRITC- labeled actin filaments in Buffer B for 3 min, rinsed with Buffer B and then incubated with Buffer E for 5 min. 100 nM fluorescent-labeled myo V in Buffer C was applied and incubated for 1 min, followed by a wash step with Buffer C containing 1 mM ATP.

### Subtilisin-treatment of microtubules

Tubulin (3.5 mg/ml) and Atto488 tubulin at a 50∶1 ratio was polymerized in Buffer A (plus 1 mM GTP) at 36°C for 90 min. To stabilize the microtubules, 20 µM taxol (Invitrogen, Darmstadt, Germany) was added, followed by an incubation at 36°C for 40 min. The C-terminal ends of α- and β-tubulin (E-hook) were removed by incubation (36°C for 45 min) of 0.6 mg/ml with subtilisin A (#P5380) at 1∶0.8 ratio in Buffer A (plus 20 µM taxol). The reaction was stopped by the addition of 2 mM PMSF (dissolved in isopropanol) and incubation at room temperature for 10 min. A previous study [12] used a nine- to twenty-three-fold higher concentration, which in our hands not only removes E-hooks but also affects microtubule integrity. Subtilisin-treated microtubules were pelleted at 27,000×g for 25 min. Pellets were washed with and resuspended in Buffer A (plus 20 µM taxol and 1 mM GTP). Samples were resolved on a 12% SDS-PAGE with subsequent Coomassie-stain or Western Blot analysis.

### Western blot analysis

After gel electrophoresis, proteins were transferred onto a nitrocellulose membrane (Protran-Whatman, Dassel, Germany) using a semi-dry transfer apparatus (Peq Lab, Erlangen, Germany). Incubation with primary (anti α- and β-tubulin) and secondary (anti-rat IgG and anti-mouse IgG) antibodies was performed overnight at 4°C and at room temperature for 1 h, respectively. Monoclonal anti-α-Tubulin (clone YL 1/2) was a kind gift from Prof. Schleicher, M. (Ludwig-Maximilians University, Munich, Germany) and monoclonal anti-β-Tubulin (clone SAP.4G5) was purchased from Santa Cruz Biotech., Inc. (Heidelberg, Germany).

### Steady-State ATPase assays

Microtubule- and actin-activated ATPase activity of myo V was determined in a coupled enzymatic assay [14], [52] with a final MgATP concentration of 1 mM. ATPase activity assays for myo V on microtubules were performed with 100 nM myo V in Buffer F and increasing amounts of microtubules (0–35 µM in Buffer G). ATP hydrolysis by myo V (100 nM) in the presence or absence of F-actin (0–20 µM) was examined in Buffer F. Measurements were carried out in 96-well plates (Greiner, Frickenhausen, Germany) using a spectrophotometer (Biotek, Friedrichshall, Germany) at an excitation wavelength of 340 nm and 23°C. The data was analyzed with Kaleidagraph 3.6 (Synergy Software, Reading, PA) software and fitted to the Michaelis-Menten function [53].

### Data acquisition

Single-molecule motility was observed at room temperature using a total internal reflection fluorescent microscope (IX71, Olympus Biosystems, Planegg, Germany) equipped with a Plan objective lens (100×; numerical aperture, 1.65) and linked to a front-illuminated CCD camera (C-9100, Hamamatsu Photonics, Herrsching, Germany). Fluorophores were excited with a solid-state laser at wavelengths of 532 or 488 nm. The optical resolution was 160 nm per 2×2 -binned pixel, the integration time 200 ms. Typically, 350 images were recorded for a total of 70 s.

### Image and data analysis

Diffusion events were defined as follows: Those events in which the myo V moves on microtubules in both directions (for >300 nm) were classified as diffusive events.

For the characterization of myo V diffusion on microtubules, the following parameters were determined: (*i*) *maximum speed* of diffusion for a given encounter was defined as the maximum displacement along the microtubule during one frame interval (i.e., 5 frames per s); (*ii*) s*can distance* for a given encounter was calculated as the distance between the two extreme positions of the microtubule, on which the respective myo V molecule has diffused along; (*iii*) *association time (t_A_)* was defined as the total time an individual myo V molecule spent on the microtubule during recording. The individual values for *t_A_* of the respective myo V constructs were obtained from an exponential fit as described [20].

The mean square displacement (MSD) for all diffusion events was calculated as described in [54], plotted as a function of time and fitted to linear function.

Single displacements of individual diffusion events for a given myo V construct were plotted as a displacement histogram. A single Gaussian was fitted to the data according to equation 1.
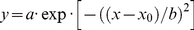
(1)Based on the obtained Gaussian fit curve for the respective displacement histogram, the variance *σ* = b^2^ was calculated. From these data, the diffusion coefficient (*D*) was determined according to the *1st law of diffusion*-derived equation, *D* = *σ*/2*t*, where *t* is the time interval between successive images.

Kymographs of representative movie -sequences from the performed single-molecule TIRFM assays on actin and microtubules were generated with the MultipleKymograph macro for IMAGEJ.

Quantification of microtubule association and diffusion was carried out with the data obtained from the performed single-molecule experiments on microtubules. For each microtubule, the numbers of diffusing and stationary particles during a 70-seconds period were counted. The numbers of diffusing and stationary particles on microtubule lengths of 1000–3000 µm were summed and divided by the corresponding microtubule lengths and time of measurement. By this procedure, the number of associated and diffusing myo V particles per microtubule unit length and time at the respective salt concentration (25, 50, 100 and 200 mM KCl) was obtained. Quantification on S-microtubules is based on data obtained from experiments in 25 mM KCl.

On actin, only events of individual myo V molecules (n≥25) walking along the filaments with an interaction time ≥2 s were classified as processive. The length of a processive run was determined manually with CellR software (Olympus Biosystems, Planegg, Germany). Velocities and runlength distributions were obtained from the single Gaussian fit according to equation 1 and the single exponential fit (as for *t_A_* of myo V on microtubules), respectively.

Distances and single displacements were measured by brightest centroid tracking, using IMAGEJ. Single-displacement boxplots as well as all other data plotting and fitting, but also the statistical analysis was performed with IgorPro software (WaveMetrics, Inc., Portland, OR).

## Supporting Information

Figure S1 **Movement and activity of myosin V** ***Wildtype*** **and loop 2 mutants on actin filaments.** Velocity (A) and runlength (B) distributions of myo V *Wildtype* were plotted as histograms. Data were obtained from single-molecule TIRFM experiments, where 100 nM Cy3-labeled myo V was incubated with Atto488-labeled F-actin in 25 mM KCl and 1 mM ATP. In (A) the data was fitted to a single Gaussian (according to equation 1, Methods section), yielding a mean velocity of 0.23 µm/s (n = 62) for *Wildtype*. For the runlength distribution in (B) an exponential curve was fitted to the histograms (solid line), resulting in a mean value of 1.41 µm (n = 62) for *Wildtype*. (C) Actin-activated ATPase for myo V *Wildtype* (black, open circles), *Minus4* (blue, open diamonds) and *Minus13* (green, open triangles) were measured with the NADH-coupled assay and plotted as a function of actin concentration (myo V concentration, 100 nM). The data were fitted to the Michaelis-Menten equation to determine the maximum ATPase rate (k_cat_) and the actin concentration at which myo V is activated half-maximally (K_m_). Data shown is representative and was reproducible. (D) Kymograph of representative motions of single Cy3-labeled myo V *Wildtype* on Atto488-labeled F-actin in buffer containing 25 mM KCl and 1 mM ATP. On actin, no movement for *Minus4* and *Minus13* was observed and hence no histograms (A and B) or kymographs (D) are depicted. All data obtained from A–C are summarized in Table S1.
(TIF)

Figure S2 **Diffusive motion of myosin V constructs on microtubules.** (A) Box-Whisker plot of the diffusion-derived displacement distribution for myo V on microtubules. Upon the analysis of TIRFM movie sequences of single myo V molecules on microtubules in 25 mM KCl, single displacements between successive image frames were determined (Figure 2B). The displacement distribution of the respective myo V constructs (as indicated) is plotted as box-whisker plot, where the top and bottom of the boxes indicate the 75 and 25 percentile, the whiskers indicate the 90 and 10 percentile, while the solid line within the boxes represents the median. As expected for one-dimensional diffusion motions, no net displacement for the respective constructs was observed and hence all respective median values center at zero. (B–D) The mean-squared displacement (MSD) data of myo V *Wildtype*, *Minus4* and *Minus13* is plotted versus time, with the individual slopes providing an estimate of the respective *D*-values. The following *D*-values were calulated: *D_Wt_* = 0.11 µm^2^/s (±0.004 µm^2^/s S.D.), *D_Minus4_* = 0.06 µm^2^/s (±0.002 µm^2^/s S.D.) and *D_Minus13_* = 0.07 µm^2^/s (±0.002 µm^2^/s S.D.). Data were obtained from single-molecule TIRFM experiments with 100 nM Cy3-labeled myo V on Atto488-labeled microtubules in 25 mM KCl. Error bars represent the S.E.M. of the squared displacement values. Diffusive motion of myo V constructs on microtubules. Color-code: myo V *Wildtype* (black), *Minus4* (blue) and *Minus13* (green).
(TIF)

Figure S3 **Interaction lifetime of diffusing myosin V on microtubules.** (A–D) The distribution of the association times (*t_A_*) for myo V *Wildtype*, *Minus4* and *Minus13* were plotted as histograms. Data were obtained from single-molecule TIRFM experiments with 100 nM Cy3-labeled myo V on Atto488-labeled microtubules in 25 mM KCl. Exponential curves fitted to the respective histograms (solid lines) yield mean *t_A_*-values of 14.1 s (n = 31), 23.8 s (n = 35) and 16.2 s (n = 32) for myo V *Wildtype* (A), *Minus4* (B) and *Minus13* (C), respectively.
(TIF)

Figure S4 **One-dimensional diffusion behavior of myosin V** ***Wildtype*** **on microtubules lacking the E-hook.** (A and B) Data were obtained from single-molecule TIRFM experiments with 100 nM Cy3-labeled myo V *Wildtype* on subtilisin-treated microtubules (Atto488-labeled) in 25 mM KCl. (A) The distribution of the values for *t_A_* is plotted as histogram with an exponential curve fit (solid line), yielding a mean *t_A_*-value of 12.4 s (n = 20). (B) In this graph, the displacement distribution of myo V *Wildtype* on S-microtubules is plotted as Box-Whisker Plot, where the top and bottom of the boxes indicate the 75 and 25 percentile, the whiskers indicate the 90 and 10 percentile, while the solid line within the box represents the median. As it was observed for myo V *Wildtype* on untreated microtubules (Figure S2A), also on S-microtubules myo V *Wildtype* exhibits no net displacement during diffusion and hence the median centers at zero.
(TIF)

Table S1 **Summary of behavior of various constructs on F-actin in 25 mM KCl.** Velocities and runlengths of the single-molecule measurements on F-actin were obtained at 1 mM ATP. Values for velocity and runlength are mean ± S.E.M. from Gaussian and exponential fits to the data (Figure S1, A and B), respectively. *n* is the number of processive runs. *K_m_* represents the actin concentration at which the ATPase rate is half the maximal rate, determined from the Michaelis-Menten curve fit (Figure S1C). *k_cat_* shows the maximum rate of ATP turnover as determined from fitting the data to the Michaelis-Menten equation (Figure S1C). *n.m.*, not measurable.
(DOC)

Table S2 **Summary of microtubule association and diffusion of various constructs at increasing salt-concentrations.** Values for microtubule association were calculated as mean ± S.E.M. from the total count of microtubule-associated (stationary and diffusing) particles per unit length and time at the indicated salt-concentrations (*left column*). Among those, the diffusing fraction of motors was determined and calculated as mean ± S.E.M. of the total number of diffusing motors per unit length and time. The portion of diffusing particles is expressed in % of the total number of microtubule-associated particles (*right column*). For details of the conditions for counting see Methods. *n.a.*, not applicable.
(DOC)

Table S3 **Summary of association and diffusion of various constructs on S-microtubules.** Values for microtubule association and diffusion were calculated as described in Table S2. Data were obtained from single-molecule studies on subtilisin-treated microtubules (*S-microtubules*) in 25 mM KCl. Significance levels in association and diffusion on *S-microtubules* vs. untreated microtubules (*P>0.05, **P<0.05 and ***P<0.005, Table S2) were determined using Student's t-Test. For details of the counting conditions applied see Methods.
(DOC)

Video S1 **One-dimensional diffusion of** ***Wildtype*** **myosin V on microtubules under high ionic strength.** Cy3-labeled myosin V *Wildtype* (bright particles) was infused into a flow cell containing surface-attached Atto 488-labeled microtubules (dim filaments). Assay was performed in 100 mM KCl. Excitation wavelength was 532 nm and representative image sequences were false-colored. This movie (89 frames) was recorded at 5 frames s^−1^ and is displayed at three-fold speed. *Scale bar* represents 2 µm.
(AVI)

Video S2 **One-dimensional diffusion of** ***Wildtype*** **myosin V on microtubules.** Cy3-labeled myosin V *Wildtype* (bright particles) was infused into a flow cell containing surface-attached Atto 488-labeled microtubules (dim filaments). Assay was performed in 25 mM KCl. Excitation wavelength was 532 nm and representative image sequences were false-colored. This movie (236 frames) was recorded at 5 frames s^−1^ and is displayed at three-fold speed. *Scale bar* represents 2 µm.
(AVI)

Video S3 **One-dimensional diffusion of** ***Minus13*** **myosin V on microtubules.** Cy3-labeled myosin V *Minus13* (bright particles) was infused into a flow cell containing surface-attached Atto 488-labeled microtubules (dim filaments). Assay was performed in 25 mM KCl. Excitation wavelength was 532 nm and representative image sequences were false-colored. This movie (272 frames) was recorded at 5 frames s^−1^ and is displayed at three-fold speed. *Scale bar* represents 2 µm.
(AVI)

Video S4 **One-dimensional diffusion of** ***Wildtype*** **myosin V on microtubules lacking E-hooks.** Cy3-labeled myosin V *Wildtype* (bright particles) was infused into a flow cell containing surface-attached Atto 488-labeled subtilisin-treated microtubules (dim filaments). Assay was performed in 25 mM KCl. Excitation wavelength was 532 nm and representative image sequences were false-colored. This movie (328 frames) was recorded at 5 frames s^−1^ and is displayed at three-fold speed. *Scale bar* represents 2 µm.
(AVI)
