# Supplementary figures and images for: Diffusion of Myosin V on Microtubules: A Fine-Tuned Interaction for Which E-Hooks Are Dispensable
Source: PLoS One. 2011 Sep 26;6(9):e25473. doi: 10.1371/journal.pone.0025473 (PMC3180451; doi:10.1371/journal.pone.0025473)

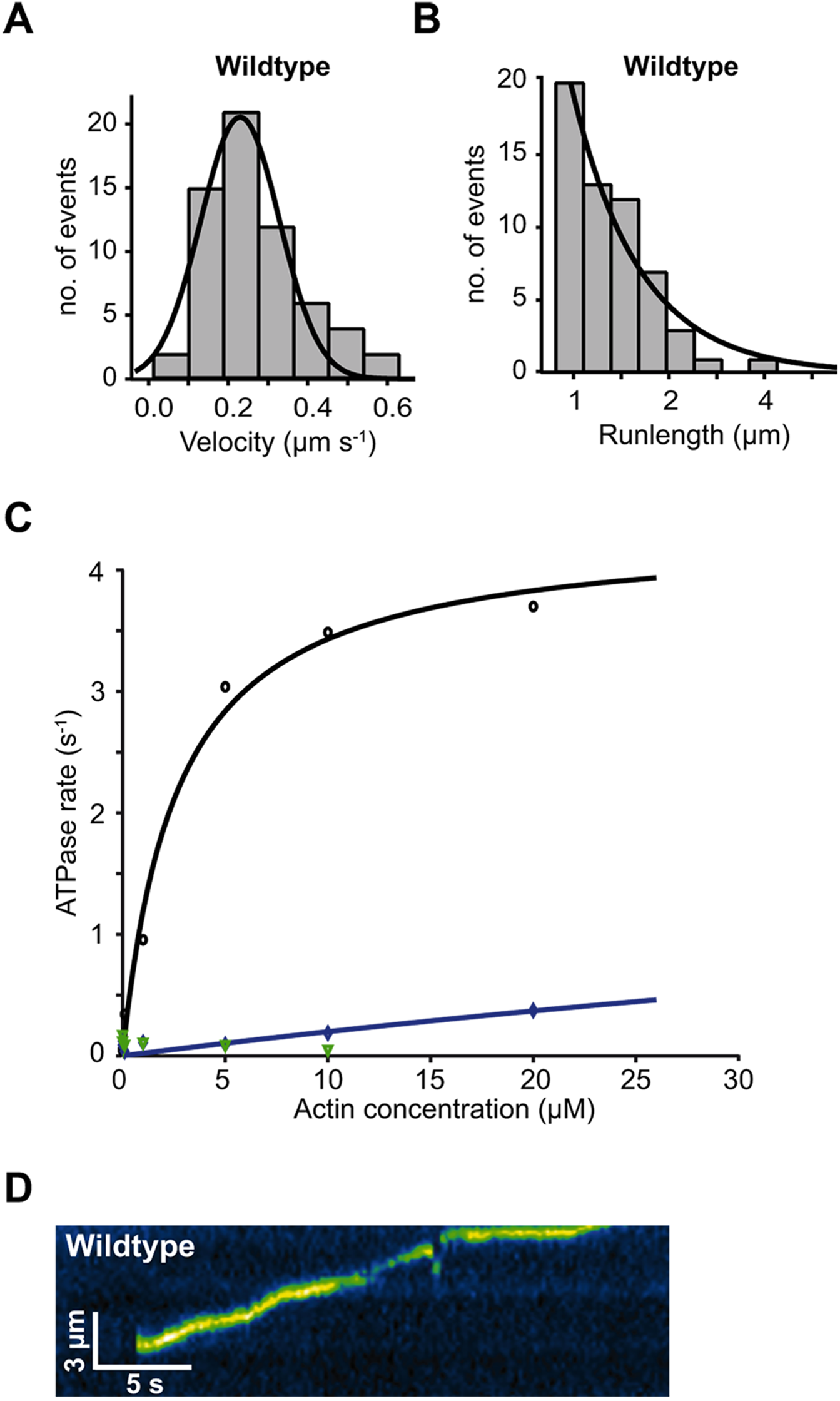

Supplement: Figure S1 — Movement and activity of myosin V Wildtype and loop 2 mutants on actin filaments. Velocity (A) and runlength (B) distributions of myo V Wildtype were plotted as histograms. Data were obtained from single-molecule TIRFM experiments, where 100 nM Cy3-labeled myo V was incubated with Atto488-labeled F-actin in 25 mM KCl and 1 mM ATP. In (A) the data was fitted to a single Gaussian (according to equation 1, Methods section), yielding a mean velocity of 0.23 µm/s (n = 62) for Wildtype. For the runlength distribution in (B) an exponential curve was fitted to the histograms (solid line), resulting in a mean value of 1.41 µm (n = 62) for Wildtype. (C) Actin-activated ATPase for myo V Wildtype (black, open circles), Minus4 (blue, open diamonds) and Minus13 (green, open triangles) were measured with the NADH-coupled assay and plotted as a function of actin concentration (myo V concentration, 100 nM). The data were fitted to the Michaelis-Menten equation to determine the maximum ATPase rate (kcat) and the actin concentration at which myo V is activated half-maximally (Km). Data shown is representative and was reproducible. (D) Kymograph of representative motions of single Cy3-labeled myo V Wildtype on Atto488-labeled F-actin in buffer containing 25 mM KCl and 1 mM ATP. On actin, no movement for Minus4 and Minus13 was observed and hence no histograms (A and B) or kymographs (D) are depicted. All data obtained from A–C are summarized in Table S1. (TIF) [file pone.0025473.s001.tif]

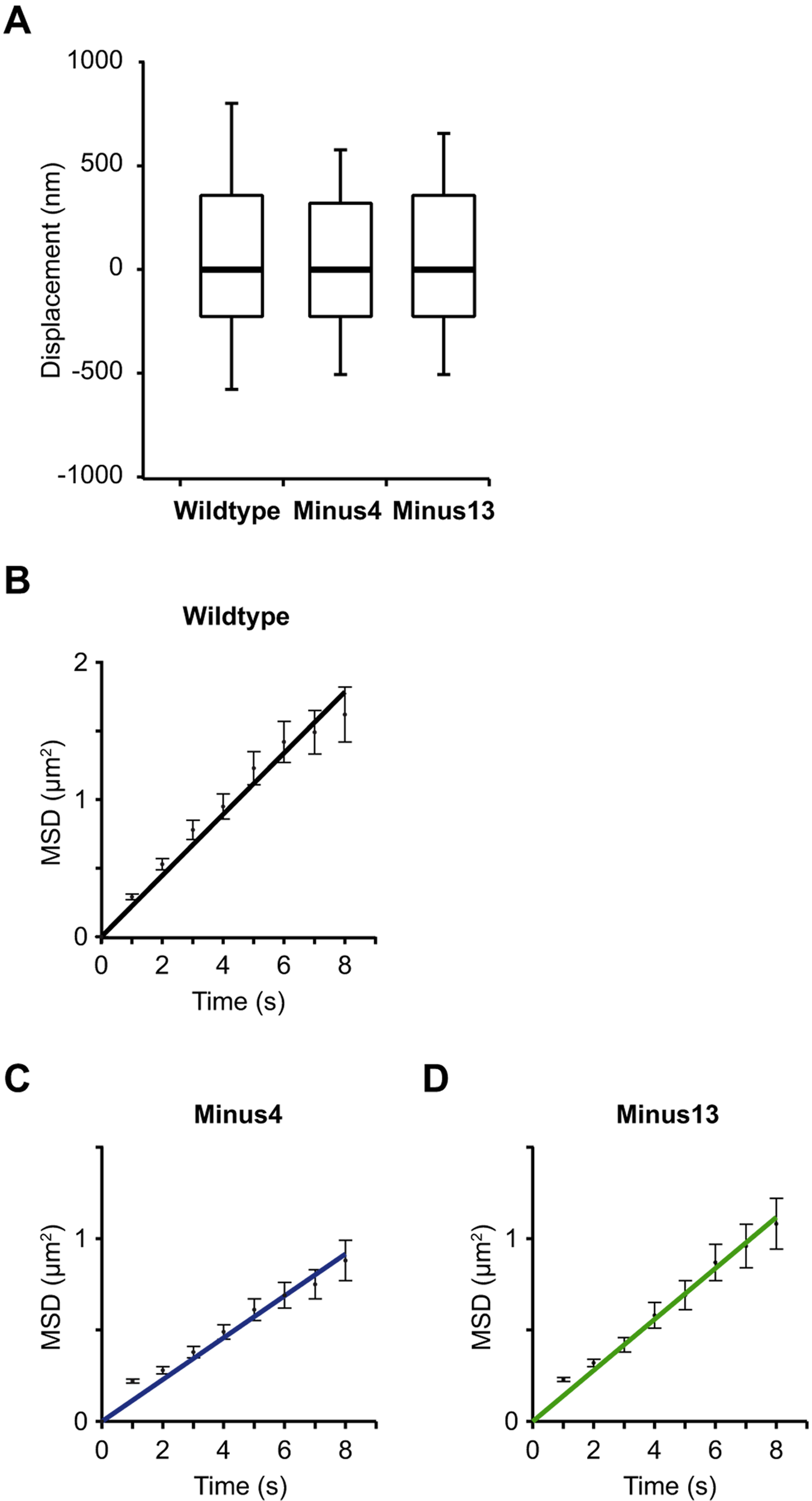

Supplement: Figure S2 — Diffusive motion of myosin V constructs on microtubules. (A) Box-Whisker plot of the diffusion-derived displacement distribution for myo V on microtubules. Upon the analysis of TIRFM movie sequences of single myo V molecules on microtubules in 25 mM KCl, single displacements between successive image frames were determined (Figure 2B). The displacement distribution of the respective myo V constructs (as indicated) is plotted as box-whisker plot, where the top and bottom of the boxes indicate the 75 and 25 percentile, the whiskers indicate the 90 and 10 percentile, while the solid line within the boxes represents the median. As expected for one-dimensional diffusion motions, no net displacement for the respective constructs was observed and hence all respective median values center at zero. (B–D) The mean-squared displacement (MSD) data of myo V Wildtype, Minus4 and Minus13 is plotted versus time, with the individual slopes providing an estimate of the respective D-values. The following D-values were calulated: DWt = 0.11 µm2/s (±0.004 µm2/s S.D.), DMinus4 = 0.06 µm2/s (±0.002 µm2/s S.D.) and DMinus13 = 0.07 µm2/s (±0.002 µm2/s S.D.). Data were obtained from single-molecule TIRFM experiments with 100 nM Cy3-labeled myo V on Atto488-labeled microtubules in 25 mM KCl. Error bars represent the S.E.M. of the squared displacement values. Diffusive motion of myo V constructs on microtubules. Color-code: myo V Wildtype (black), Minus4 (blue) and Minus13 (green). (TIF) [file pone.0025473.s002.tif]

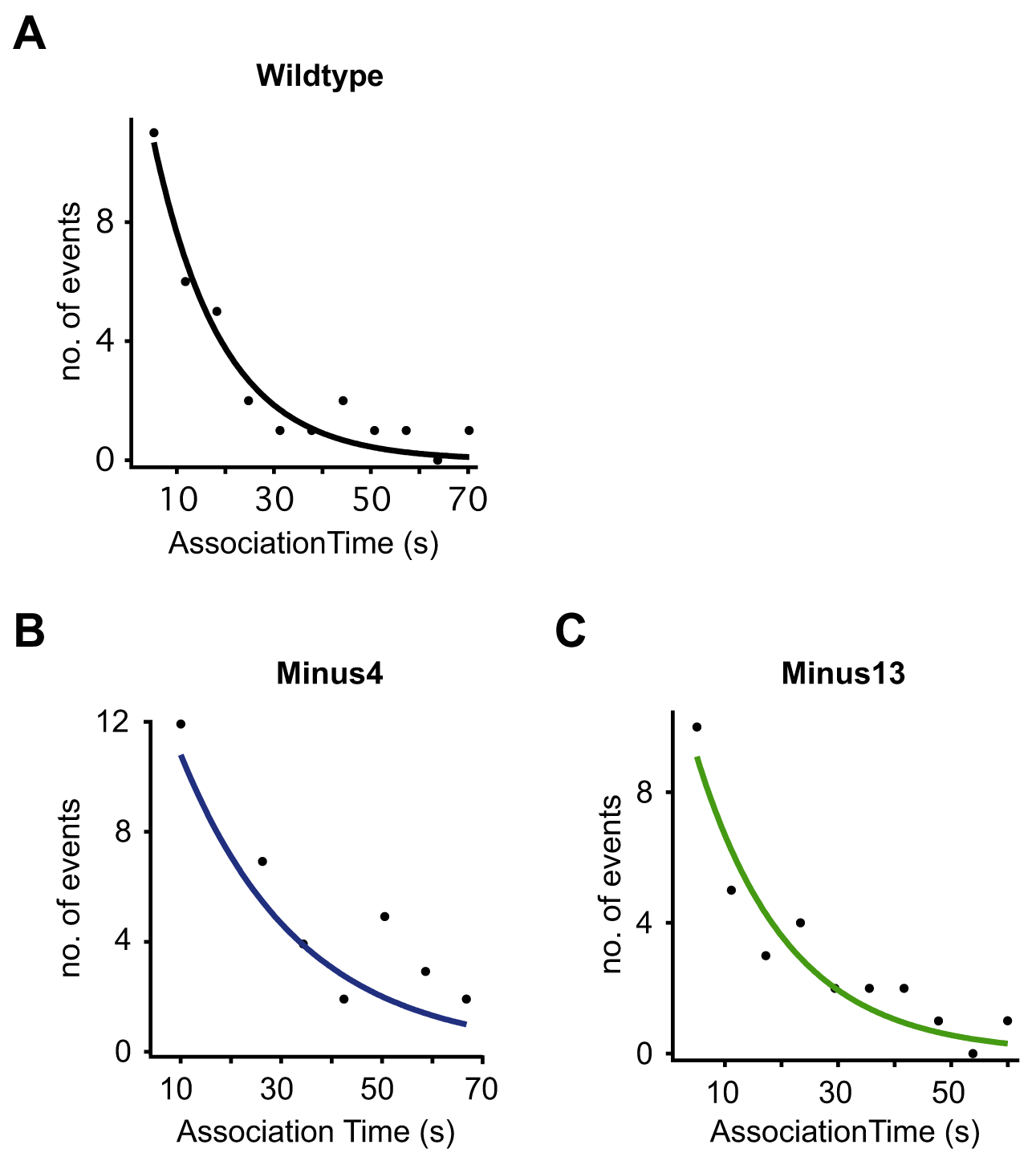

Supplement: Figure S3 — Interaction lifetime of diffusing myosin V on microtubules. (A–D) The distribution of the association times (tA) for myo V Wildtype, Minus4 and Minus13 were plotted as histograms. Data were obtained from single-molecule TIRFM experiments with 100 nM Cy3-labeled myo V on Atto488-labeled microtubules in 25 mM KCl. Exponential curves fitted to the respective histograms (solid lines) yield mean tA-values of 14.1 s (n = 31), 23.8 s (n = 35) and 16.2 s (n = 32) for myo V Wildtype (A), Minus4 (B) and Minus13 (C), respectively. (TIF) [file pone.0025473.s003.tif]

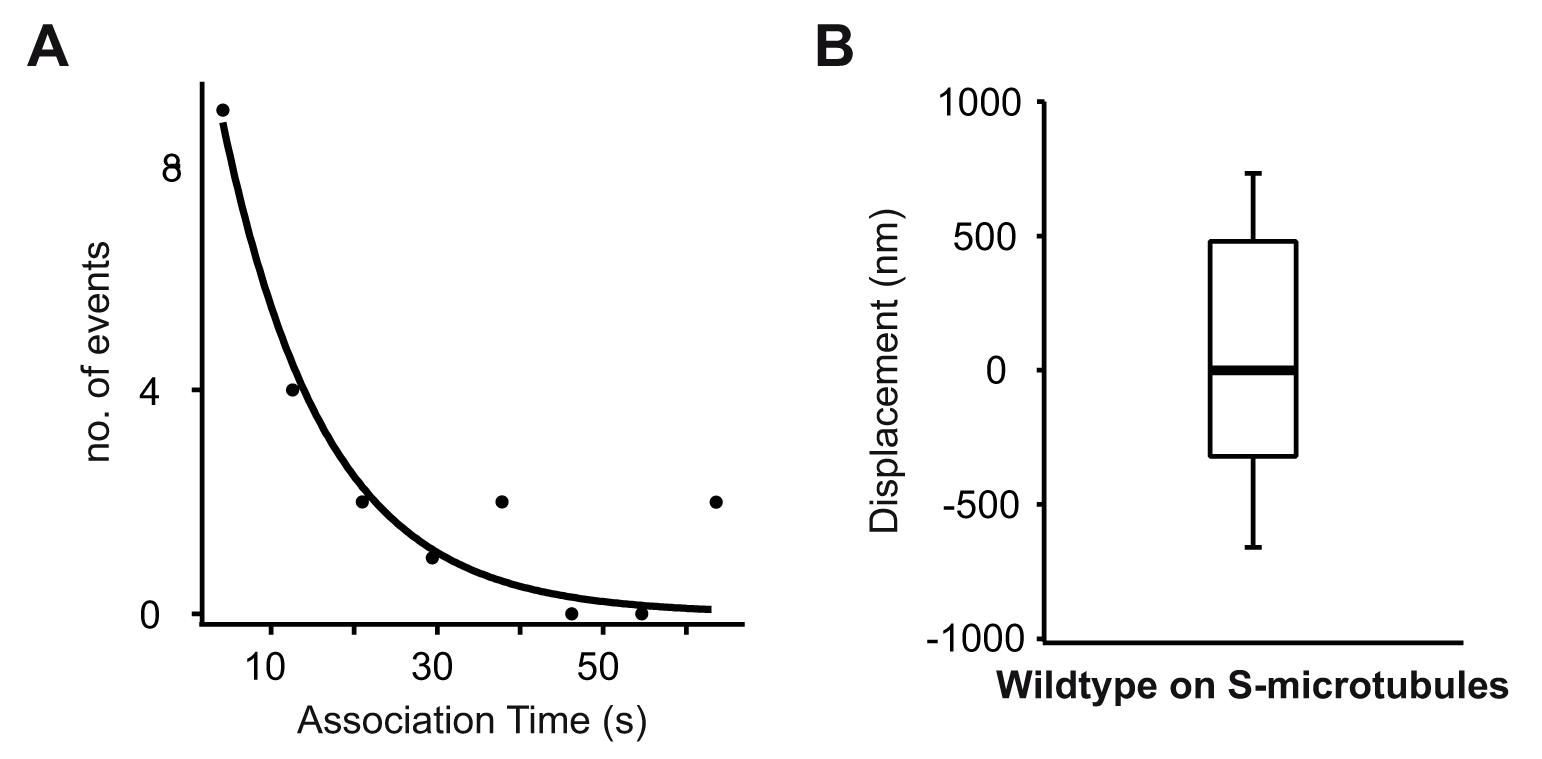

Supplement: Figure S4 — One-dimensional diffusion behavior of myosin V Wildtype on microtubules lacking the E-hook. (A and B) Data were obtained from single-molecule TIRFM experiments with 100 nM Cy3-labeled myo V Wildtype on subtilisin-treated microtubules (Atto488-labeled) in 25 mM KCl. (A) The distribution of the values for tA is plotted as histogram with an exponential curve fit (solid line), yielding a mean tA-value of 12.4 s (n = 20). (B) In this graph, the displacement distribution of myo V Wildtype on S-microtubules is plotted as Box-Whisker Plot, where the top and bottom of the boxes indicate the 75 and 25 percentile, the whiskers indicate the 90 and 10 percentile, while the solid line within the box represents the median. As it was observed for myo V Wildtype on untreated microtubules (Figure S2A), also on S-microtubules myo V Wildtype exhibits no net displacement during diffusion and hence the median centers at zero. (TIF) [file pone.0025473.s004.tif]
