# Supplementary material for: Diffusion of Myosin V on Microtubules: A Fine-Tuned Interaction for Which E-Hooks Are Dispensable
Source: PLoS One. 2011 Sep 26;6(9):e25473. doi: 10.1371/journal.pone.0025473 (PMC3180451; doi:10.1371/journal.pone.0025473)
Supplement: Table S1 — Summary of behavior of various constructs on F-actin in 25 mM KCl. Velocities and runlengths of the single-molecule measurements on F-actin were obtained at 1 mM ATP. Values for velocity and runlength are mean ± S.E.M. from Gaussian and exponential fits to the data (Figure S1, A and B), respectively. n is the number of processive runs. Km represents the actin concentration at which the ATPase rate is half the maximal rate, determined from the Michaelis-Menten curve fit (Figure S1C). kcat shows the maximum rate of ATP turnover as determined from fitting the data to the Michaelis-Menten equation (Figure S1C). n.m., not measurable. (DOC) [file pone.0025473.s005.doc]

**Table S1. Summary of behavior of various constructs on F-actin in 25 mM KCl.**

|  | Velocity  (nm s^-1^) | Runlength  (µm) | n | K_m_  (µM) | k_cat_  (mol of ATP s^-1^ **⋅** (mol of myosin)^-1^) |
| --- | --- | --- | --- | --- | --- |
| MyoV (Wildtype) | 230 ± 4 | 1.41 ± 0.04 | 62 | 2.6 | 4.3 |
| MyoV (Minus4) | n.m. | n.m. | n.m. | n.m. | n.m. |
| MyoV (Minus13) | n.m. | n.m. | n.m. | n.m. | n.m. |
