# Supplementary material for: Diffusion of Myosin V on Microtubules: A Fine-Tuned Interaction for Which E-Hooks Are Dispensable
Source: PLoS One. 2011 Sep 26;6(9):e25473. doi: 10.1371/journal.pone.0025473 (PMC3180451; doi:10.1371/journal.pone.0025473)
Supplement: Table S2 — Summary of microtubule association and diffusion of various constructs at increasing salt-concentrations. Values for microtubule association were calculated as mean ± S.E.M. from the total count of microtubule-associated (stationary and diffusing) particles per unit length and time at the indicated salt-concentrations (left column). Among those, the diffusing fraction of motors was determined and calculated as mean ± S.E.M. of the total number of diffusing motors per unit length and time. The portion of diffusing particles is expressed in % of the total number of microtubule-associated particles (right column). For details of the conditions for counting see Methods. n.a., not applicable. (DOC) [file pone.0025473.s006.doc]

**Table S2. Summary of microtubule association and diffusion of various constructs at increasing salt-concentrations.**

|  | Ionic strength condition  (c_(KCl)_ in mM) | Microtubule associations  (particles **⋅ (**mm min)^-1^) | MyoV diffusions  (particles **⋅ (**mm min)^-1^) | Portion of  Myo V diffusion events  (% of total MT associations) |
| --- | --- | --- | --- | --- |
| MyoV (Wildtype) | 25 | 55.4 ± 5.0 | 17.6 ± 3.7 | 31.7 |
| MyoV (Minus4) |  | 51.3 ± 8.6 | 7.0 ± 2.4 | 13.6 |
| MyoV (Minus13) |  | 12.8 ± 2.5 | 2.1 ± 1.3 | 16.7 |
| MyoV (Wildtype) | 50 | 45.9 ± 6.1 | 4.7 ± 1.7 | 10.3 |
| MyoV (Minus4) |  | 33.4 ± 5.0 | 4.3 ± 1.8 | 12.9 |
| MyoV (Minus13) |  | 13.0 ± 3.3 | 1.8 ± 1.1 | 13.9 |
| MyoV (Wildtype) | 100 | 10.9 ± 2.5 | 0.4 ± 0.4 | 3.5 |
| MyoV (Minus4) |  | 25.2 ± 5.2 | 0.0 ± 0.0 | 0.0 |
| MyoV (Minus13) |  | 5.6 ± 2.0 | 0.8 ± 0.8 | 14.6 |
| MyoV (Wildtype) | 200 | 6.4 ± 2.6 | 0.6 ± 0.6 | 9.7 |
| MyoV (Minus4) |  | 11.2 ± 3.7 | 0.0 ± 0.0 | 0.0 |
| MyoV (Minus13) |  | 0.0 ± 0.0 | 0.0 ± 0.0 | n.a. |
