# Supplementary material for: Diffusion of Myosin V on Microtubules: A Fine-Tuned Interaction for Which E-Hooks Are Dispensable
Source: PLoS One. 2011 Sep 26;6(9):e25473. doi: 10.1371/journal.pone.0025473 (PMC3180451; doi:10.1371/journal.pone.0025473)
Supplement: Table S3 — Summary of association and diffusion of various constructs on S-microtubules. Values for microtubule association and diffusion were calculated as described in Table S2. Data were obtained from single-molecule studies on subtilisin-treated microtubules (S-microtubules) in 25 mM KCl. Significance levels in association and diffusion on S-microtubules vs. untreated microtubules (*P>0.05, **P<0.05 and ***P<0.005, Table S2) were determined using Student's t-Test. For details of the counting conditions applied see Methods. (DOC) [file pone.0025473.s007.doc]

**Table S3. Summary of association and diffusion of various constructs on S-microtubules.**

|  | Microtubule associations  (particles **⋅ (**mm min)^-1^) | MyoV diffusions  (particles **⋅ (**mm min)^-1^) | Portion of  Myo V diffusion events  (% of total MT associations) |
| --- | --- | --- | --- |
| MyoV (Wildtype) | 58.2 ± 6.7 * | 8.5 ± 2.8 ** | 12.5 |
| MyoV (Minus4) | 65.0 ± 6.9 * | 0.0 ± 0.0 *** | 0.0 |
| MyoV (Minus13) | 50.8 ± 6.5 *** | 0.0 ± 0.0 * | 0.0 |
